# Fractal scaling of particle-size distribution and associations with soil properties of Mongolian pine plantations in the Mu Us Desert, China

**DOI:** 10.1038/s41598-017-06709-8

**Published:** 2017-07-27

**Authors:** Jifeng Deng, Jinghao Li, Ge Deng, Hangyong Zhu, Ruohan Zhang

**Affiliations:** 1 College of Forestry, Shenyang Agricultural University, Shenyang, 110866 P.R. China; 2 Research Station of Liaohe-River Plain Forest Ecosystem, Chinese Forest Ecosystem Research Network (CFERN), Shenyang Agricultural University, Shenyang, 110866 P.R. China; 3 Harbin City Forestry Academy, Harbin, 150028 P.R. China; 4 Faculty of Regional Environment Science, Tokyo University of Agriculture, Tokyo, 156-8502 Japan

## Abstract

Mongolian pine plantations (MPPs) composed of *Pinus sylvestris* var. *mongolica* (*P*. *sylvestris*) are used for desertification control and restoration of degraded land in arid and semi-arid regions. We studied soil changes associated with *P*. *sylvestris* by comparing top (0–20 cm) and sub-top (20–40 cm) soil properties across 8 stand density gradients of MPPs ranging from 900 ± 5–2700 ± 50 trees ha^–1^. The study was conducted in the uncovered Sandy Land in the southern Mu Us Desert, China. The relationships between the volume fractal dimensions (*D*) of soil particle size distribution and soil physicochemical properties were evaluated. *D* was determined using a laser diffraction technique and soil properties were measured. In the top layer, *P*. *sylvestris* significantly positively affected soil physicochemical properties except for bulk density and total nitrogen. These effects were not observed in the sub-top soil layer. *D* values ranged from 1.52 ± 0.29–2.08 ± 0.06 and were significantly correlated with stand density. Significant correlations were observed between *D* and soil properties (except total nitrogen) in the top soil layer. Given these results, we concluded that *D* is a sensitive and useful index because it quantifies changes in soil properties that additionally implies desertification in the studied area.

## Introduction

Overcultivation, urbanization, and adverse climate variations, such as droughts and floods can result in the degradation of arid and semi-arid lands^1–3^. China has large areas of desertification (approximately 2.64 billion ha) because of overpopulation and insufficient natural resources^2^. Among the numerous desert areas, the Mu Us Desert in northern China is the places most seriously affected by desertification^4, 5^. The Mu Us Desert is located on the southern Ordos Plateau and lies at the northern margin of the Asian summer monsoon^4^. The Mu Us Desert covers an area of approximately 4 million ha and is an important part of the farming and pastoral zone of China^5^. Desertification in the Mu Us Desert is primarily evident in the transformation of formerly anchored dunes into semi-anchored and mobile dunes^5^.

Vegetation cover loss and subsequent desertification results in degradation of several soil physicochemical properties^6^. Numerous means and methods, such as introducing mechanical sand barriers^7^, biological soil crust^8^, and afforestation have been carried out in an effort to restore soil fertility and modify sand areas. Afforestation is considered the most effective method for reducing wind damage and increasing biodiversity. For more than 50 years, through environmental management, afforestation has been used to control desertification and increase timber production in Sandy areas^2, 9^.

Mongolian pines are an important species grown on Sandy Lands. Mongolian pines are a variety of Scots pine (*P*. *sylvestris* var*. mongolica*) that is naturally distributed in the Daxinganling mountains, Haila’er, Wangong, Cuogang, He’erhongde, Hunhe, and Ha’erhahe areas in the Inner Mongolian Autonomous Region and Hulunbeier Sandy plain of China (50°10′–53°33′N, 121°11′–127°10′E) and parts of Russia and Mongolia (46°30′–53°59′N, 118°00′–130°08′E). Mongolian pines grow at altitudes of 600–2000 m^10^ and have excellent wind sheltering, cold and drought resistance, and broad climate adaptability. They have been introduced from the Hulunbei’er Sandy Land to many other parts of China, particularly through the “Three-North” Protective Forest System Project^10^. The creation of Mongolian pine plantations (MPPs) was aimed to improve the Sandy Lands, reduce desertification, and increase timber supply^11^. Revegetation projects were initiated in Mu Us Desert in the mid-1950s. In the earliest projects, MPPs were planted in Yulin City, Shaanxi Province, which is located in the southern Mu Us Desert. The MPPs adapted well to the area compared with those in the original native regions of northern China^11^.

Although Mongolian pines were successfully grown on Sandy Lands by seedling plantings, difficulties such as slow growth, withered tops, and abnormal development, affected plantings that were made before the late 1980 s^2–6^. These problems raised concerns about the management of MPPs. Studies have demonstrated that the main reasons for failure of MPPs were habitat changes, physical structures of soil, and loss of soil nutrients^2, 8, 11–14^.

Sustained nutrient availability for plant growth and controlling the cycling of nutrients in living communities are basic tenets in ecosystem management^15, 16^. Extensive changes in plantations affect the dynamics of soil physicochemistry. MPPs problems are also caused by soil nutrient deficiency. The interactions between *P. sylvestris*, soil erosion, and desertification have been thoroughly studied^1, 2, 6, 9–11^. MPPs can improve and restore ecosystem balance, including physical, biological, and biogeochemical processes^17, 18^. However, an understanding of the soil properties in MPPs and their interrelations is limited. The effects of stand densities on soil properties would also benefit from further research.

Soil organization and functions can be characterized using single parameters but it is unclear if this is the optimal approach in monitoring soil degradation and desertification. Use of individual fractions (such as analysis of clay and finer fractions) or using soil organic carbon (SOC) are commonly employed to characterize soil quality. However, soil is a complex system in which many biological and physical components interact across space and time scales^19, 20^. Between the 2 aforementioned approaches, individual fractions typically de-emphasize coarse fractions and emphasize fine particles. Textual analysis cannot provide complete information and this analysis results in a waste of soil data. Furthermore, the results are unsuitable for evaluating real soil systems such as desert soils that contain a large proportion of coarse particles^21^. Although SOC is widely used in soil quality assessment, this method is insensitive to environmental change over shorter time scales^22^. These traditional methodologies therefore cannot provide complete information and quantitatively represent fundamental attributes by use of a practical index. By contrast, fractal measures can use all soil particle-size distribution (PSD) information, including clay, silt, and sand particle data^23^. PSD is used in soil classification and the estimation of soil hydraulic properties, such as soil water retention curves, soil hydraulic conductivity, and soil bulk density (BD)^24–27^. Different PSD-driven sorption properties of soil affect the mineralization of decoupled carbon and nitrogen, as well as the activity of invertase and xylanase during organic matter decomposition^28–30^. Therefore, PSD is useful for understanding the physical and chemical processes of soil water and the development of soil nutrient cycles^31^. The volumetric distribution of soil particles is usually replaced by the mass distribution of soil particles when evaluating the soil fractal dimension^32^. However, the density of soil particles with different radii varies^33^. Therefore, the soil particle volumetric distribution can be used to directly calculate the soil volume fractal dimension (*D*). Laser diffraction is a useful technique that has been used to measure soil *D*, and it is a reliable method for estimating PSD^34^. The use of soil *D* is a new approach to describe the distribution of soil particles. Significant linear correlations have been found between *D* and various soil properties using this technique^21, 23^. The method permits quantifying and integrating information on the biological, chemical, and physical characteristics of soil measured on different depths scales^31^.

Much additional information on the mutual relationships of MPPs and soil properties is needed. An effective index for quantifying MPPs effects on soil properties in desert areas should also be developed. This study evaluated soil status dynamics in forest ecosystems, particularly the effects that different stand densities of MPPs have on soil properties. We hypothesized that topsoil (0–40 cm) properties are affected by MPPs establishment and stand densities. Changes in top (0–20 cm) and sub-top (20–40 cm) soil properties were studied across a population density gradient of MPPs and in the referenced uncovered Sandy Land (CK) in Yulin City, Shaanxi Province (located in the southern Mu Us Desert, Northern China). The specific objectives were as follows: (1) to determine how changes in topsoil properties, including *D* and physicochemical properties vary with different stand densities of MPPs; and (2) to evaluate the possibility that *D* of soil PSD can be used as a practical index for quantifying variations in soil physicochemical properties and the implications of desertification. This study may improve the design and management of afforestation by using MPPs that increase soil nutrients and improve the physical structure of soil. These changes would also be beneficial to stand development.

## Results

### PSD and fractal characteristics of topsoil properties in different MPPs

Table 1 shows the soil PSD in the different soil sampling plots, including the CK. Sand particles (50–2000 μm diameter) are the dominant soil particle class, and account for >70% of the total PSD. Clay (<2 μm) and silt (2–50 μm) contents were significantly lower than sand particles. The clay contents were less than 4.00% of total PSD.

**Table 1:** Variations of PSD and *D* values for different densities of MPPs and CK plots.

Pn	Layer	PSD (%)			*D*
		Clay (0–2 μm)	Silt (2–50 μm)	Sand (50–2000 μm)	
P_I_	Top	3.32 ± 0.84	23.87 ± 0.78	72.80 ± 3.21	2.01 ± 0.07
	Sub-top	3.54 ± 0.47	19.20 ± 0.39	77.26 ± 2.65	2.08 ± 0.06
P_II_	Top	1.34 ± 1.32	24.91 ± 1.34	73.75 ± 2.70	2.00 ± 0.05
	Sub-top	2.64 ± 0.41	19.57 ± 0.41	77.79 ± 3.48	2.07 ± 0.06
P_III_	Top	1.26 ± 1.20	21.87 ± 1.45	76.88 ± 4.32	1.89 ± 0.06
	Sub-top	1.44 ± 0.81	18.32 ± 0.82	80.23 ± 3.62	2.06 ± 0.09
P_IV_	Top	1.16 ± 0.98	21.78 ± 0.99	77.06 ± 3.25	1.70 ± 0.34
	Sub-top	2.93 ± 0.87	15.13 ± 0.87	81.94 ± 2.98	2.04 ± 0.06
P_V_	Top	1.10 ± 0.14	19.47 ± 1.15	79.43 ± 2.15	1.68 ± 0.29
	Sub-top	2.29 ± 0.32	14.04 ± 0.33	83.67 ± 3.26	2.00 ± 0.08
P_VI_	Top	1.75 ± 0.09	16.46 ± 0.11	81.79 ± 2.02	1.62 ± 0.25
	Sub-top	1.14 ± 0.67	13.00 ± 0.68	85.86 ± 4.00	1.96 ± 0.06
P_VII_	Top	0.72 ± 0.84	14.89 ± 0.79	84.39 ± 3.21	1.58 ± 0.32
	Sub-top	1.56 ± 0.14	8.41 ± 0.13	90.03 ± 1.02	1.94 ± 0.06
P_VIII_	Top	2.13 ± 0.05	13.14 ± 0.03	84.73 ± 2.00	1.52 ± 0.29
	Sub-top	0.88 ± 0.73	9.72 ± 0.72	89.40 ± 2.36	1.94 ± 0.12
CK	Top	1.18 ± 0.76	11.32 ± 0.76	87.50 ± 3.22	1.42 ± 0.25
	Sub-top	2.32 ± 0.73	6.36 ± 0.74	91.32 ± 4.32	1.71 ± 0.29

Data are means ± standard error (n = 3).

In MPPs, clay and silt contents gradually increased with stand density. Compared with CK (1.18 ± 0.76% (top) and 2.32 ± 0.73% (sub-top), and 11.32 ± 0.76% (top) and 6.36 ± 0.74% (sub-top) for clay and slit contents separately) from P_I_ (3.32 ± 0.84% (top) and 3.54 ± 0.47% (sub-top), 23.87 ± 0.78% (top) and 19.20 ± 0.39% (sub-top)) to P_VIII_ (2.13 ± 0.05% (top) and 0.88 ± 0.73% (sub-top), 13.14 ± 0.03% (top) and 9.72 ± 0.72% (sub-top)), clay contents increased by as much as 182.26% and 52.15% for the top and sub-top layers, and by 80.64% for the top layer. Silt contents increased by as much as 110.94% and 201.98%, and by 16.22% and 52.92% for the top and sub-top layers, respectively. As a result, clay and silt content differences between MPPs and CK were high. Furthermore, sand particle content from P_VIII_ to P_I_ decreased. Compared with CK, sand particle content in P_I_ and P_VIII_ decreased by 20.19% (top) and 18.19% (sub-top), and by 3.27% (top) and 2.15% (sub-top) respectively. Meanwhile, sand content within the same plot increased from the top to sub-top layer, in addition to a decrease in silt and clay (expect P_I_, P_II_, P_III_, P_IV_, P_V_, and P_VII_) contents. In contrast, clay contents of CK were increased with increasing soil depth.

*D* values were subsequently calculated with Eq. 1 based on the PSD data. The *D* values for the different plots are shown in Table 1. *D* of soil PSD ranged from 1.52 ± 0.29–2.01 ± 0.07 (top) and from 1.94 ± 0.12–2.08 ± 0.06 (sub-top) (except CK, which was 1.42 ± 0.25 and 1.71 ± 0.29 for the top and sub-top layers). Although there was a slight change in the value of *D* between MPPs, with increasing stand densities of MPPs, *D* values increased gradually. The *D* values of all MPPs were generally higher than CK in all topsoil layers. *D* values in the sub-top layer of all plots were higher than that of the top layers. Soils with greater clay and silt contents had higher *D* values, whereas soils with a greater amount of sand particles had lower *D* values (Table 1).

### Physical properties of soil subsections in different MPPs

**Figure 1:**
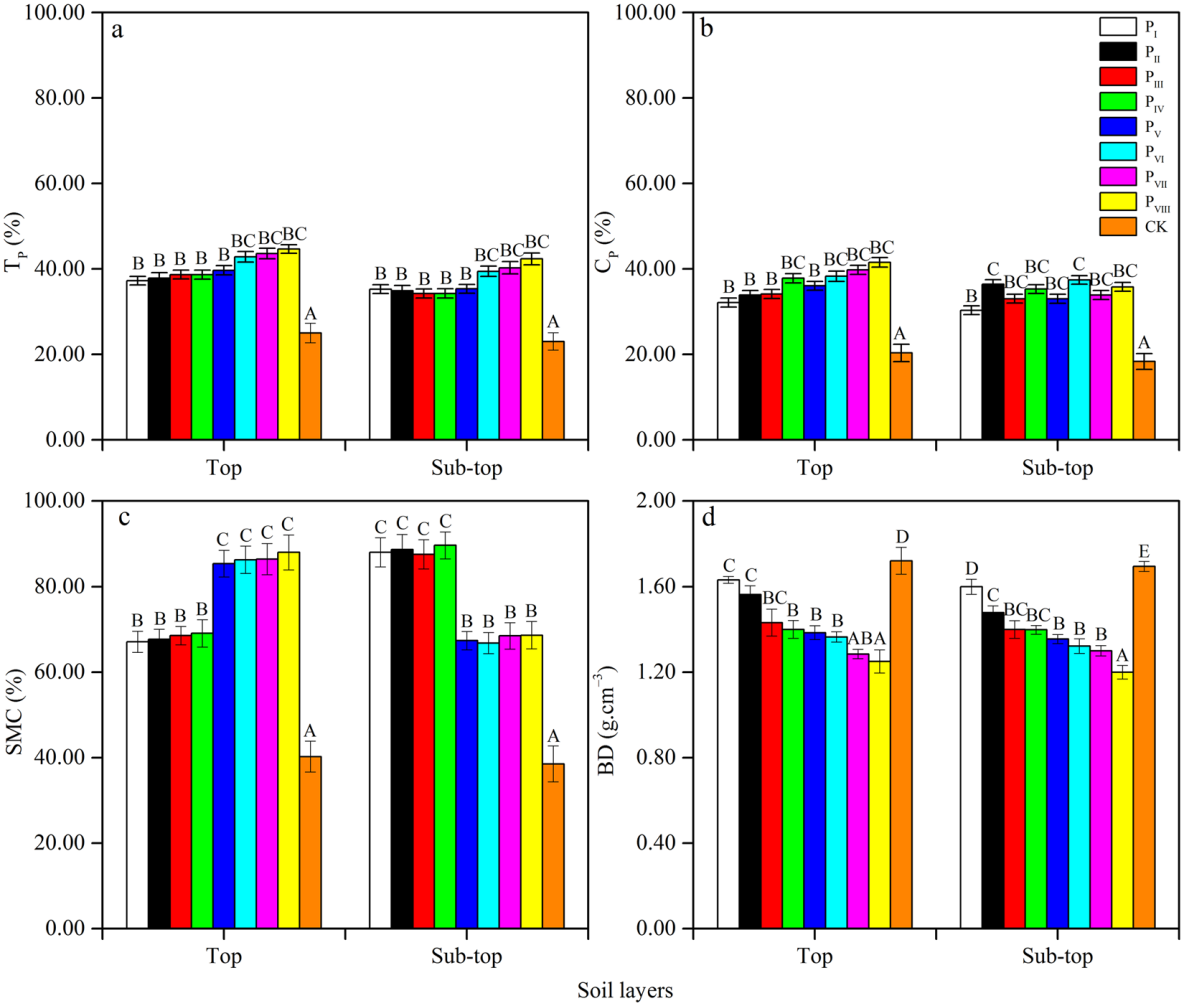
Variations of soil physical properties (T_P_ (**a**), C_P_ (**b**), SMC (**c**), and BD (**d**)) in different MPPs and CK plots. Vertical bars indicate standard errors of means (n = 3). ANOVA with a LSD test was used with different letter in the same row are significantly different at the 0.05 level.

No significant variations in soil total porosity (T_P_) were noted among any of the MPPs in both top and sub-top layers (*p* > 0.05) (Fig. 1a). A significant difference was only observed between CK and MPPs. Capillary porosity (C_P_), saturated soil moisture content (SMC), and BD showed significant differences in all layers among all MPPs (*p* < 0.05) (Fig. 1b-d). P_V_, P_VI_, P_VII_, and P_VIII_ had higher T_P_, C_P_, and SMC, and lower BD values compared with other plots in the top layer (*p* < 0.05). Meanwhile, P_VI_, P_VII_ and P_VIII_ had the lowest SMC, which ranged from 66.81 ± 2.45%–68.66 ± 3.21% in the sub-top layer. The CK soil had the lowest T_P_, C_P_ and SMC, and had the highest BD values, which were 25.00 ± 2.30% (top) and 23.00 ± 2.02% (sub-top), 20.31 ± 2.01% (top) and 18.32 ± 1.86% (sub-top), 40.24 ± 3.62% (top) and 38.53 ± 4.21% (sub-top), and 1.72 ± 0.06 g.cm^−3^ (top) and 1.70 ± 0.02 g.cm^−3^ (sub-top).

A clear tendency to increase or decrease from high stand density (P_I_) to low stand density (P_VIII_) was apparent, which was the opposite of C_P_ in the MPPs (Fig. 1). Among all plots, T_P_ ranged from 37.24 ± 1.00%–44.65 ± 1.00% (top) and 35.22 ± 1.03%–42.31 ± 1.36% (sub-top); C_P_ ranged from 32.11 ± 1.06%–41.51 ± 1.10% (top) and 30.30 ± 1.02%–35.76 ± 1.02% (sub-top); SMC ranged from 67.11 ± 2.45%–88.03 ± 4.10% (top) and 66.81 ± 2.45%–89.68 ± 3.14% (sub-top); and BD ranged from 1.28 ± 0.02 g.cm^−3^–1.63 ± 0.02 g.cm^−3^ (top) and 1.30 ± 0.02–1.60 ± 0.04 g.cm^−3^ (sub-top). With increasing soil depth, T_P_ and C_P_ averages decreased 8.47% and 6.86% in the same plot, whereas the BD average increased 2.45%.

T_P_, C_P_, SMC, and BD were significantly correlated with each other in the top layer (correlation coefficients ranged from 0.79–0.94, *p* < 0.01). In the sub-top layer, T_P_, SMC, and BD were significantly correlated with each other (correlation coefficients ranged from 0.75–0.77, *p* < 0.01); however, C_P_ was not significantly correlated with SMC or BD (*p* > 0.05) (Table 2).

**Table 2:** Pearson analysis of soil *D* and soil physical properties for different densities of MPPs plots.

Traits	Layer	*D*	T_P_	C_P_	SMC	BD
*D*	Top	1	−0.89^**^	−0.95^**^	−0.88^**^	0.95^**^
	Sub-top	1	−0.88^**^	−0.44	0.92^**^	0.90^**^
T_P_	Top		1	0.91^*^	0.89^**^	−0.88^**^
	Sub-top		1	0.39	−0.77^*^	−0.76^*^
C_P_	Top			1	0.79^*^	−0.94^**^
	Sub-top			1	−0.28	−0.55
SMC	Top				1	−0.81^*^
	Sub-top				1	0.75^*^
BD	Top					1
	Sub-top					1

^*^Correlation is significant at the 0.05 level (2-tailed). ^**^Correlation is significant at the 0.01 level (2-tailed).

### SOC and soil nutrients of soil subsections in different MPPs

**Figure 2:**
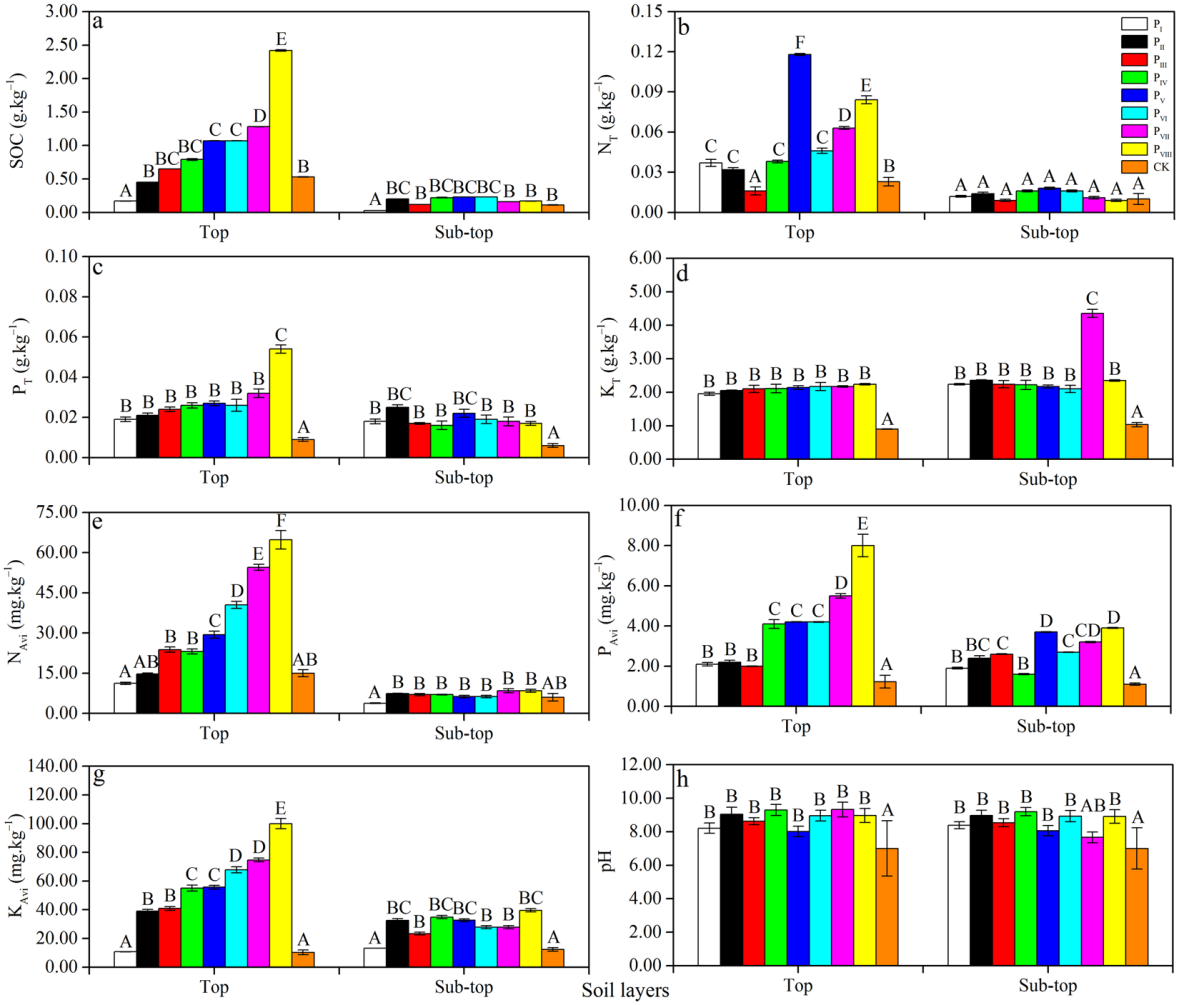
Variations in SOC (**a**) and soil nutrients (N_T_ (**b**), P_T_ (c), K_T_ (**d**), N_avi_ (**e**), P_avi_ (**f**), and K_avi_ (**g**)) in MPPs and CK plots. Data are means ± standard error (n = 3). Means with the different letter in the same layers are significantly different at the *p* = 0.05 (LSD test).

Together with the positive changes in soil physical structure, SOC and soil nutrients increased (Fig. 2). Compared with the CK, SOC and soil nutrients were higher in the MPPs. In the top layer, the SOC and soil nutrients increased as the stand density decreased. Such effects were clear and had significant regularity and large variation amplitude. Except for soil total nitrogen (N_T_), P_VIII_ had highest SOC, soil total phosphorus (P_T_), soil total potassium (K_T_), soil available nitrogen (N_Avi_), soil available phosphorus (P_Avi_), and soil rapid available potassium (K_Avi_) values at 2.42 ± 0.01 g.kg^−1^, 0.05 ± 0.002 g.kg^−1^, 2.24 ± 0.02 g.kg^−1^, 64.80 ± 3.45 mg.kg^−1^, 8.00 ± 0.56 mg.kg^−1^, and 100.00 ± 3.62 mg.kg^−1^, respectively, which differed significantly from CK (0.53 ± 0.0032 g.kg^−1^, 0.009 ± 0.0009 g.kg^−1^, 0.90 ± 0.01 g.kg^−1^, 15.00 ± 1.32 mg.kg^−1^, 1.23 ± 0.32 mg.kg^−1^, and 10.33 ± 1.65 mg.kg^−1^, respectively) and P_I_ (0.17 ± 0.001 g.kg^−1^, 0.02 ± 0.001 g.kg^−1^, 1.95 ± 0.04 g.kg^−1^, 11.20 ± 0.41 mg.kg^−1^, 2.10 ± 0.09 mg.kg^−1^, and 10.70 ± 0.02 mg.kg^−1^, respectively) (*p* < 0.05). Meanwhile, in the sub-top layer, no trend was followed. However, compared with the MPPs, CK had the lowest SOC and soil nutrients (0.11 ± 0.004 g.kg^−1^, 0.01 ± 0.004 g.kg^−1^, 0.006 ± 0.0009 g.kg^−1^, 1.03 ± 0.06 g.kg^−1^, 6.00 ± 1.36 mg.kg^−1^, 1.10 ± 0.06 mg.kg^−1^, and 12.36 ± 1.24 mg.kg^−1^, respectively).

Changes in SOC and soil nutrients varied significantly, particularly the levels of SOC (from P_I_ to P_VIII_, the values were 0.17 ± 0.001 g.kg^−1^, 0.45 ± 0.001 g.kg^−1^, 0.65 ± 0.001 g.kg^−1^, 0.79 ± 0.01 g.kg^−1^, 1.07 ± 0.001 g.kg^−1^, 1.07 ± 0.001 g.kg^−1^, 1.28 ± 0.001 g.kg^−1^, and 2.42 ± 0.01 g.kg^−1^, respectively), N_T_ (from P_I_ to P_VIII_, 0.04 ± 0.003 g.kg^−1^, 0.03 ± 0.001 g.kg^−1^, 0.02 ± 0.003 g.kg^−1^, 0.04 ± 0.0001 g.kg^−1^, 0.12 ± 0.00007 g.kg^−1^, 0.05 ± 0.002 g.kg^−1^, 0.06 ± 0.0001 g.kg^−1^, and 0.08 ± 0.003 g.kg^−1^, respectively) and N_Avi_ (from P_I_ to P_VIII_, 11.20 ± 0.41 mg.kg^−1^, 14.70 ± 0.41 mg.kg^−1^, 23.80 ± 0.98 mg.kg^−1^, 23.10 ± 0.97 mg.kg^−1^, 29.40 ± 1.32 mg.kg^−1^, 40.50 ± 1.32 mg.kg^−1^, 54.50 ± 1.11 mg.kg^−1^, and 64.80 ± 3.45 mg.kg^−1^, respectively) in the top layer (Fig. 2a,b,e), and the levels of P_Avi_ (from P_I_ to P_VIII_, 1.90 ± 0.04 mg.kg^−1^, 2.40 ± 0.12 mg.kg^−1^, 2.60 ± 0.021 mg.kg^−1^, 1.60 ± 0.03 mg.kg^−1^, 3.70 ± 0.02 mg.kg^−1^, 2.70 ± 0.01 mg.kg^−1^, 3.20 ± 0.03 mg.kg^−1^, and 3.90 ± 0.02 mg.kg^−1^, respectively) and K_Avi_ (from P_I_ to P_VIII_, 13.26 ± 0.01 mg.kg^−1^, 32.5 ± 1.22 mg.kg^−1^, 23.40 ± 1.00 mg.kg^−1^, 34.90 ± 1.10 mg.kg^−1^, 32.70 ± 1.00 mg.kg^−1^, 27.90 ± 1.10 mg.kg^−1^, 27.90 ± 1.10 mg.kg^−1^, and 39.60 ± 1.10 mg.kg^−1^, respectively) in the sub-top layer (Fig. 2f,g). Further, except for K_T_, SOC and other soil nutrients within the same stand density of MPPs significantly decreased from the top to sub-top layer (*p* < 0.05) (Fig. 2d). Moreover, the pH values among all MPPs were higher at 8.80 ± 0.34 (top) and 8.58 ± 0.30 (sub-top) than the CK, which were 7.00 ± 1.65 (top) and 7.00 ± 1.24 (sub-top) (Fig. 2h). Soil properties did not include any acidic conditions.

In addition, in the top layer, SOC had significantly positive correlations with P_T_, K_T_, N_avi_, P_avi_, and K_avi_, and correlation coefficients were 0.97, 0.90, 0.93, 0.96, and 0.95, respectively (*p* < 0.01). However, in the sub-top layer, SOC and soil nutrients were not significantly correlated (*p* > 0.05) (Table 3).

**Table 3:** Pearson analysis of soil *D* and SOC, soil nutrients for different densities of MPPs plots.

Traits	Layer	*D*	SOC	N_T_	P_T_	K_T_	N_avi_	P_avi_	K_avi_
*D*	Top	1	−0.85^**^	−0.60	−0.74^*^	−0.92^**^	−0.89^**^	−0.89^**^	−0.92^**^
	Sub-top	1	−0.45	0.03	0.21	−0.44	−0.57	−0.72^*^	−0.52
SOC	Top		1	0.58	0.97^**^	0.90^**^	0.93^**^	0.96^**^	0.95^**^
	Sub-top		1	0.63	0.32	−0.09	0.52	0.28	0.79^*^
N_T_	Top			1	0.50	0.51	0.49	0.62	0.50
	Sub-top			1	0.43	−0.32	−0.29	−0.17	0.18
P_T_	Top				1	0.79^*^	0.88^**^	0.94^**^	0.87^**^
	Sub-top				1	−0.11	−0.02	0.15	0.12
K_T_	Top					1	0.89^**^	0.84^**^	0.97^**^
	Sub-top					1	0.48	0.23	−0.02
N_avi_	Top						1	0.93^**^	0.94^**^
	Sub-top						1	0.50	0.76^*^
P_avi_	Top							1	0.92^**^
	Sub-top							1	0.47
K_avi_	Top								1
	Sub-top								1

^*^Correlation is significant at the 0.05 level (2-tailed). ^**^Correlation is significant at the 0.01 level (2-tailed).

### Relationship between *D* and soil physicochemical properties of soil subsections in different MPPs

**Figure 3:**
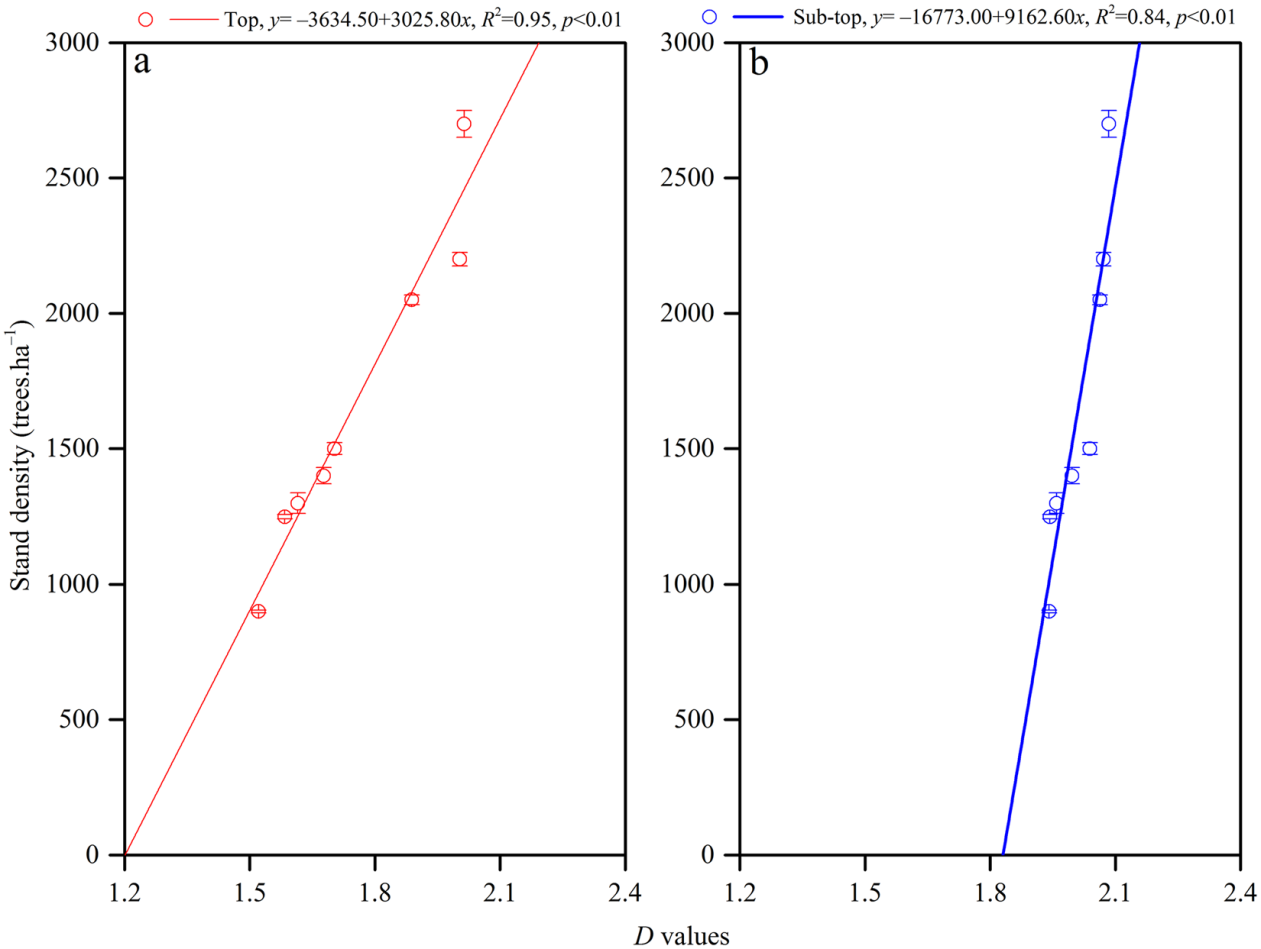
Relationships between *D* values and stand density of MPPs at the top layer (**a**) and sub-top layer (**b**).

**Figure 4:**
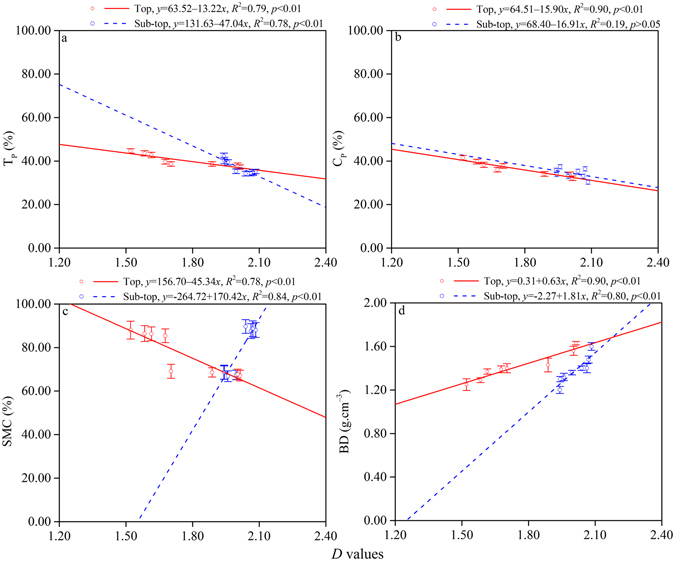
Relationships between *D* values and T_P_ (**a**), C_P_ (**b**), SMC (**c**), BD (**d**) of MPPs at the top layer and sub-top layer.

**Figure 5:**
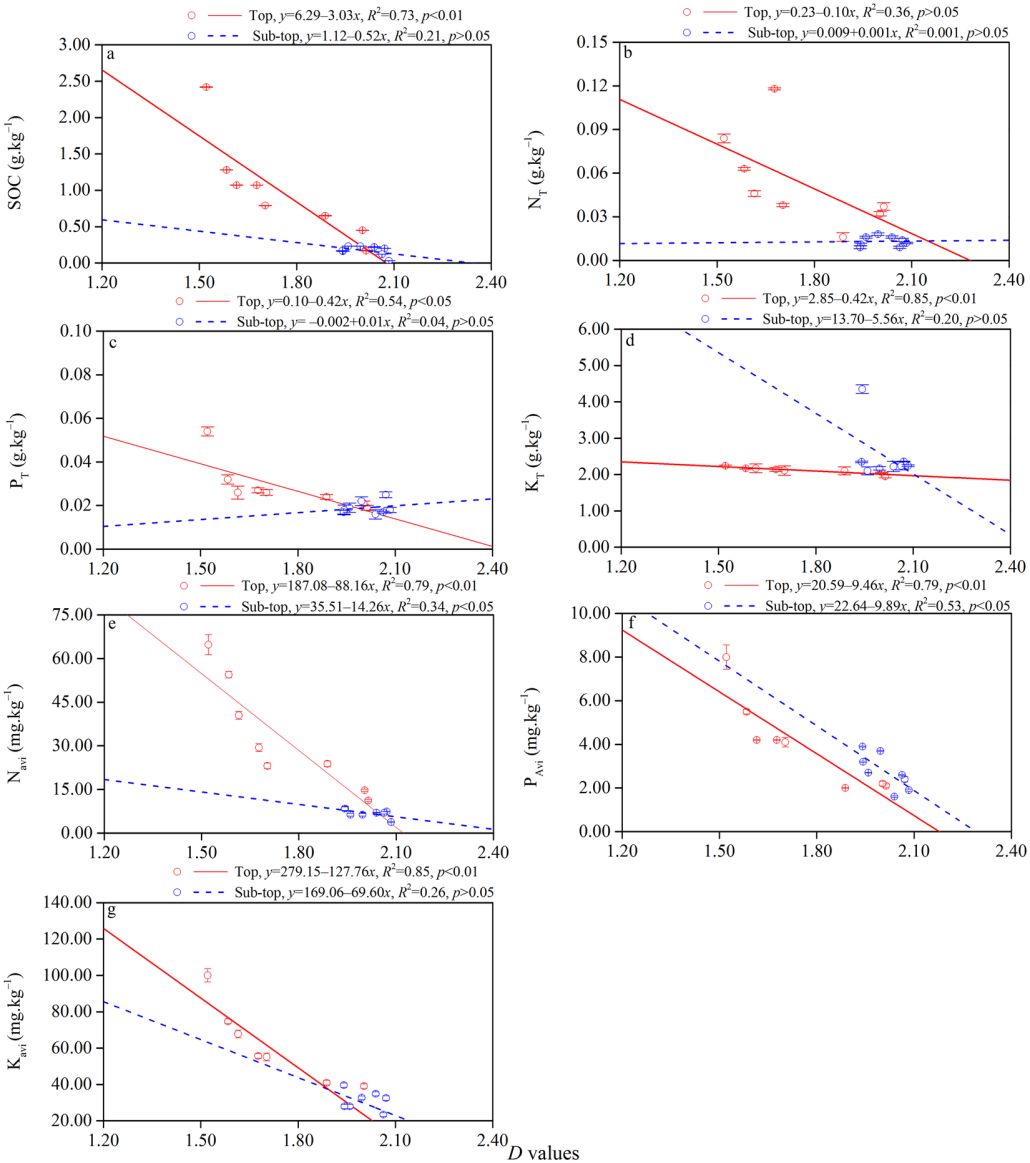
Relationships between *D* values and SOC (**a**), N_T_ (**b**), P_T_ (**c**), K_T_ (**d**), N_avi_ (**e**), P_avi_ (**f**), K_avi_ (**g**) of MPPs at the top layer and sub-top layer.

Linear regression and correlation analysis were used to study the relationships between *D* and stand density, physical soil properties including T_P_, C_P_, SMC, and BD, and chemical soil properties including SOC and selected soil nutrients (Figs 3, 4 and 5; Tables 2 and 3). The results showed positive linear correlation between *D* values and stand density (top *R* ^2^ = 0.95, *p* < 0.01; sub-top *R* ^2^ = 0.84, *p* < 0.01). Furthermore, the *D* values were more affected by the top soil layer (Fig. 3).

A significant negative linear correlation was found between T_P_, SMC, and *D* values with *R* ^2^ ranging from 0.78–0.79, *p* < 0.01 (Fig. 4a,c). Lack of a significant correlation was noted between C_P_ and *D* values in the sub-top layer, with *R* ^2^ = 0.19 (*p* > 0.05) (Fig. 4b). In contrast a positive linear correlation exists between SMC (sub-top), BD and *D* values (*R* ^2^ ranged from 0.80–0.90, *p* < 0.01) (Fig. 4c,d). This reverse correlation and the different variations in BD, T_P_, and C_P_ were mutually verified. Pearson analysis results indicated strong correlations between soil *D* and selected soil physics properties (Table 2). *D* was significantly positively correlated with BD, and significantly negatively correlated with T_P_, C_P_, and SMC in the top layer. The correlation coefficients were 0.95, −0.89, −0.95, and −0.88, respectively (*p* < 0.01). *D* was significantly positively correlated with SMC and BD, and negatively correlated with T_P_ and C_P_ in the sub-top layer. The correlation coefficients were 0.92 and 0.90 (*p* < 0.01), and −0.88 (*p* < 0.01) and −0.44 (*p* > 0.05), respectively. Soil *D* was more strongly affected by BD in the top layer and SMC in the sub-top layer.

Figure 5 shows the relationship between *D* values and SOC and soil nutrients. *D* had significant negative linear correlation with SOC (top), P_T_ (top), K_T_ (top), N_Avi_ (top), P_Avi_ (top and sub-top), and K_Avi_ (top) with *R* ^2^ = 0.73, 0.57, 0.85, 0.79, 0.79, 0.53, and 0.85, respectively (*p* < 0.05). However, no significant correlation existed between *D* values and SOC (sub-top), N_T_ (top and sub-top), P_T_ (sub-top), K_T_ (sub-top), N_Avi_ (sub-top), and K_Avi_ (sub-top) (*p* > 0.05). In addition, the Pearson analysis indicated that *D* values were not related to N_T_. In the top layer, SOC, P_T_, K_T_, N_avi_, P_avi_, and K_avi_ had the strongest influence on *D*, and correlation coefficients were −0.85, −0.74, −0.92, −0.89, −0.89, and −0.92, respectively. Meanwhile, in the sub-top layer, only P_avi_ strongly influenced *D*, and the correlation coefficient was −0.72 (*p* < 0.01) (Table 3).

## Discussion

We investigated the effect of MPPs on topsoil properties and tested the feasibility of soil *D* as an indicator of soil property variation in the process of desert evolution. Consequently, the level of soil degradation and desertification in southern Mu Us Desert could be determined. Our main findings and analyses are discussed as follows.

### Effects of MPPs on topsoil physicochemical properties

Plants affect soil properties, which in turn alter plant growth and interspecific competition. This process establishes a plant-soil feedback system^35–39^. Many physicochemical properties of soil, such as T_P_, C_P_, BD, SOC, N, P, K, and pH, are mainly determined by plant type and cover^36^. Soil plays an important role in the fertility and stability of forest ecosystems by supporting microorganism communities, which release nutrients necessary for vegetation development and improve the physical structure of the soil^40^. We found that soil physicochemical properties are improved by MPPs. These forests can protect the Sandy soil surface from wind erosion. For example, soil particles and dusts in airstreams are largely blocked by trees and undergrowth shrubs. Erosive force and carriage capability are absorbed by MPPs^41^. MPPs soil physical structure had good permeability, and nutrient losses due to wind erosion in the topsoil of CK were significantly higher than in the MPPs (Figs 1 and 2).

Our findings are consistent with those of Huang *et al*.^42^, who found that the expansion of drylands, unprotected land, and erosion-induced land degradation may increase the extent of desertification. This expansion can also lead to SOC storage reduction and CO_2_ emissions into the atmosphere, which contribute to global warming and form a positive feedback cycle. The Mu Us Desert has a typical arid and semi-arid continental monsoonal climate. The enhanced warming of arid and semi-arid areas will contribute to their degradation. Enhanced surface warming in drylands can be explained by surface processes^43^. In drylands, low soil moisture content limits evaporation and limited vegetation cover leads to low transpiration rates and C loss^44^. Vegetation can lower air temperature via transpiration^45^ and by converting absorbed sunlight into chemical energy via photosynthesis to fix C^46^. This reduces the extra heating from increased greenhouse gases and results in lowered warming rates. We found that the presence of MPPs has a positive effect on topsoil properties, which is significant for managing the impact of climate warming on unprotected land. The C concentration in the topsoil decreased significantly in the CK compared to the Mu Us Lands with MPPs. This observation is consistent with previous observations on this semi-arid area^47, 48^ and other afforested sites^49^. Loss of soil C in the CK has been attributed to the effect of decreased organic matter inputs. Our data supports this mechanism since the C concentration in all particle-size fractions and in aggregates decreased in bare Sandy Land. These results are qualified with the observation that changes in BD may influence the interpretation of the C storage differences in BD values among MPPs and CK plots were large (see Fig. 1), with lower values in the CK and highest values in MPPs. In addition, compared to the CK, the increase in topsoil C in MPPs was associated with an increase in C concentration in both silt and sand particle-size fractions, and these increases were coincident with a decrease in the coarse sand fraction (Table 1). This decline in soil C stock might be ameliorated by adoption of improved afforestation practices. Thus, efforts should be made to retain as much plant cover as possible.

In previous studies, several processes were found to influence net C storage following pine afforestation of the Sandy Lands. As the forest grows, net C accumulation could occur from increased litter production and protection of soil organic matter by physical or biotic mechanisms^50^. Soil organic matter dynamics have been linked to changes in soil physical structure, especially aggregate formation^51^. To enhance soil C storage during afforestation of Sandy soils in semi-arid regions, disruption of vegetation should be minimized during the planting stage. These results are the same as those by Chen *et al*.^52^, who conducted research on organic carbon in soil physical fractions under different-aged plantations of Mongolian pine in the semi-arid region of Northeast China.

Our results are also consistent with those of a previous study conducted in the semi-arid Horqin Sandy Land of northern China^14^. The afforestation of areas with active sand dunes using MPPs had positive effects on SOC, N, and P accumulation in the plants and soil. Additionally, the greatest improvement of soil SOC and selected soil nutrients occurred in the upper soil layer after plantation establishment^14^.

Soil physical properties differ among topsoil layers, and these differences may affect precipitation infiltration and evaporation^53^. In the present study, sub-top soil layers had larger particle sizes (greater proportion of sand particles) than top soil layers (see Table 1), allowing for more rapid movement into deep soil layers. The results agree with those of Dai *et al*.^54^ showing that the spatial variability of soil particle size and porosity result in differences in soil properties.

In the MPPs study area, the spatial pattern of SOC, soil P_T_, K_T_, N_avi_, P_avi_, and K_avi_ distribution was consistent with distribution of T_P_ and C_P_, suggesting the coupling of soil N, P, and K transformations, and the dependence of soil N, P, and K availability on soil water availability^55^. Water, SOC, N, P, and K are the main limiting factors for pine tree growth in the semi-arid area^56^. Regional ecosystem management must consider the availability and balance of these resources. Thus, protection of the litter layer is strongly recommended to ameliorate soil degradation and nutrient limitation in the study area since the litter layer was not only the main source of soil organic matter and available nutrients, but also a regulator of soil microbial activity^57, 58^. Some beetle species live in the litter layer, and the decomposition of their bodies provides important nutrient resources in arid and semi-arid regions^21^.

Variations in soil properties differed among the stand densities of MPPs, indicating that an optimal stand density is needed for best results. We believe that P_VIII_ (900 ± 5 trees.ha^−1^) is the optimal tree planting density. Under this density, we found the highest values of soil physicochemical properties, such as T_P_, C_P_, SOC, P_T_, K_T_, N_avi_, P_avi_, and K_avi_, whereas BD had the lowest values.

### Soil *D* as a practical indicator for desertification in MPPs

Soil texture classification is usually measured using the percentages of clay, silt, and sand within certain size ranges. Soil texture is critical for understanding the transportation and storage of soil water and nutrients, and the mineralization of organic matter content^59^. In this study of *P*. *sylvestris* plantations, the average *D* values continued to increase over time. This change led to optimal particle distribution of afforested Sandy Land compared to that of bare Sandy Land. The change was also beneficial by decreasing BD and increasing water infiltration. Such effects were more significant in the top layer of the soil profile. The strong correlation between *D* and the soil nutrients can be interpreted as being caused by an increase in fine soil particles and organic matter content. Given that soil clay particles bind nutrients in soil^60^, an increase in clay concentration enhances soil adhesive forces. Accordingly, the ability of soil to absorb water and the cation content in soil are both enhanced. Higher clay concentrations were found in MPPs soils than in CK soils. Clay is more easily eroded by runoff than sand, thereby enabling MPPs to act as a barrier to soil and wind erosion and enhancing the deposition of sediment carried by erosion processes^60^. Once the Sandy Land loses the protection of *P*. *sylvestris*, or wind velocity and precipitation exceed the threshold, accumulative fine particles can be quickly eroded and lost.

Linear regression and correlation analysis indicated that *D* values had a highly significant negative correlation with most of the selected soil properties. Fine fractions (clay and silt) are associated with fertile, hydrophilic, and biodiversity-rich soil systems; however, a different phenomenon was observed in the present study. The highest MPPs stand density (P_I _2700 ± 50 trees.ha^−1^) had the highest *D* values. This may be because artificial forests with high stand density can effectively resist wind erosion. Wind erosion causes nutrient and functional losses and transports the fine soil particles, thereby reducing the water-holding capacity, depleting soil structure, and diminishing biological properties^61–63^. Fine particle losses caused by wind-induced erosion cause land degradation and desertification^28^. In general, soil *D* is closely related to soil functions, but the 2 parameters are interdependent. Given the capability of MPPs to reduce water and wind erosion, plantations can change the process and intensity of erosion. Different stand densities of MPPs change the movement and deposition of soil, thereby causing the redistribution of soil clay. Therefore, the soil particles and *D* vary within these MPPs, and the extent to which *D* reflects changes in soil nutrient content requires further study. Ecological systems are complex, and the estimation of soil *D* in different MPPs can help determine the changes in soil properties and vulnerability to desertification. Meanwhile, low *D* values are practical for suitable stand density of MPPs.

Further, unique among other soil nutrients, soil N_T_ is an expectation. In this study, a non-significant relationship between *D* and N_T_ was observed, corresponding with irregularities in N_T_ values among the different stand densities of MPPs. Nitrogen turnover is complex because it combines nitrogen mineralization, ammonia volatilization, nitrification, and denitrification^12, 16^. In forest ecosystems, soluble organic N and inorganic N (NH_4_^+^-N and NO_3_^−^N) are the major nitrogen sources available for plant growth^11^. Plants growing on mineral soils in the temperate zone do not efficiently utilize soluble organic N for growth, so soluble organic N is rarely reported in Sandy Land areas. The amounts of available inorganic forms of N in soils are generally small. A small pool of NO_3_^−^N may indicate either a low nitrification rate, a high rate of NO_3_^−^N uptake by plants, or rapid denitrification^12^. During our study, N_T_ content in MPPs was higher than in the CK, indicating that MPPs improved N_T_ in soils, although the degree of improvement was not significant.

### Recommendations for further research

Several previous studies have proposed a combination of several physical, chemical, biological and biochemical properties as indicators of soil status^64^. Specific indicators of soil microbial activity have been proposed to assess soil status, including several enzyme activities specifically related to N, P, and C cycles, and some general microbial indicators, such as dehydrogenase activity and soil respiration^26^. However, lack of consideration for other major influencing factors and indexes, which consider both representativeness and comprehensiveness, limits the validity of these methods. Addressing the limitations of this study in future studies can provide a better understanding of soil improvement through use of xeric-adapted plant species such as *P*. *sylvestris*. This would provide guidance for more successful afforestation, combating desertification, and environmental protection in the arid and semi-arid regions of China^12, 13, 42, 65–69^.

## Conclusions

The establishment of MPPs in the Mu Us Desert positively changed the topsoil properties. Soil clay and silt particle contents, T_P_, C_P_, SMC, SOC, and soil nutrients increased in MPPs compared with those in the CK. These increases were accompanied by a decrease in soil sand particle content and BD. With a decrease in stand density, soil physicochemical properties in all MPPs plots significantly decreased. Linear regression and correlation analysis showed that the *D* values had significant linear relationships with soil physicochemical properties (except for N_T_), as well as stand densities in the top layer. *R* ^2^ values ranged from 0.54–0.95 (*p* < 0.05) and correlation coefficients ranged from 0.60–0.95 (*p* < 0.05). In the sub-top layer, the *R* ^2^ values (0.001–0.84) were lower and correlation coefficients ranged from 0.03–0.92. In summary, *D* was sensitive to soil coarsening and soil properties. Therefore, *D* can be used as a practical index to quantify changes in soil properties and indicate desertification vulnerability.

This research was limited by the omission of other soil depths and microelement levels. *P*. *sylvestris* is a shallow-rooted plant and 80% of its roots are found at 0–100 cm soil depth. Other soil nutrients, such as Ca, may have significant direct or indirect impact on plant growth and soil properties. Additionally, only 3 sampling points were used in the present study. Future studies should address these limitations.

## Materials and Methods

### Experiment site description

Mu Us Desert has an arid and semi-arid continental monsoonal climate, with an annual precipitation ranging from 200–400 mm, evaporation of 1800–2500 mm, and aridity of 1.0–2.5^70, 71^. The Mu Us Desert has a low to moderate wind-energy environment^72^.

**Figure 6:**
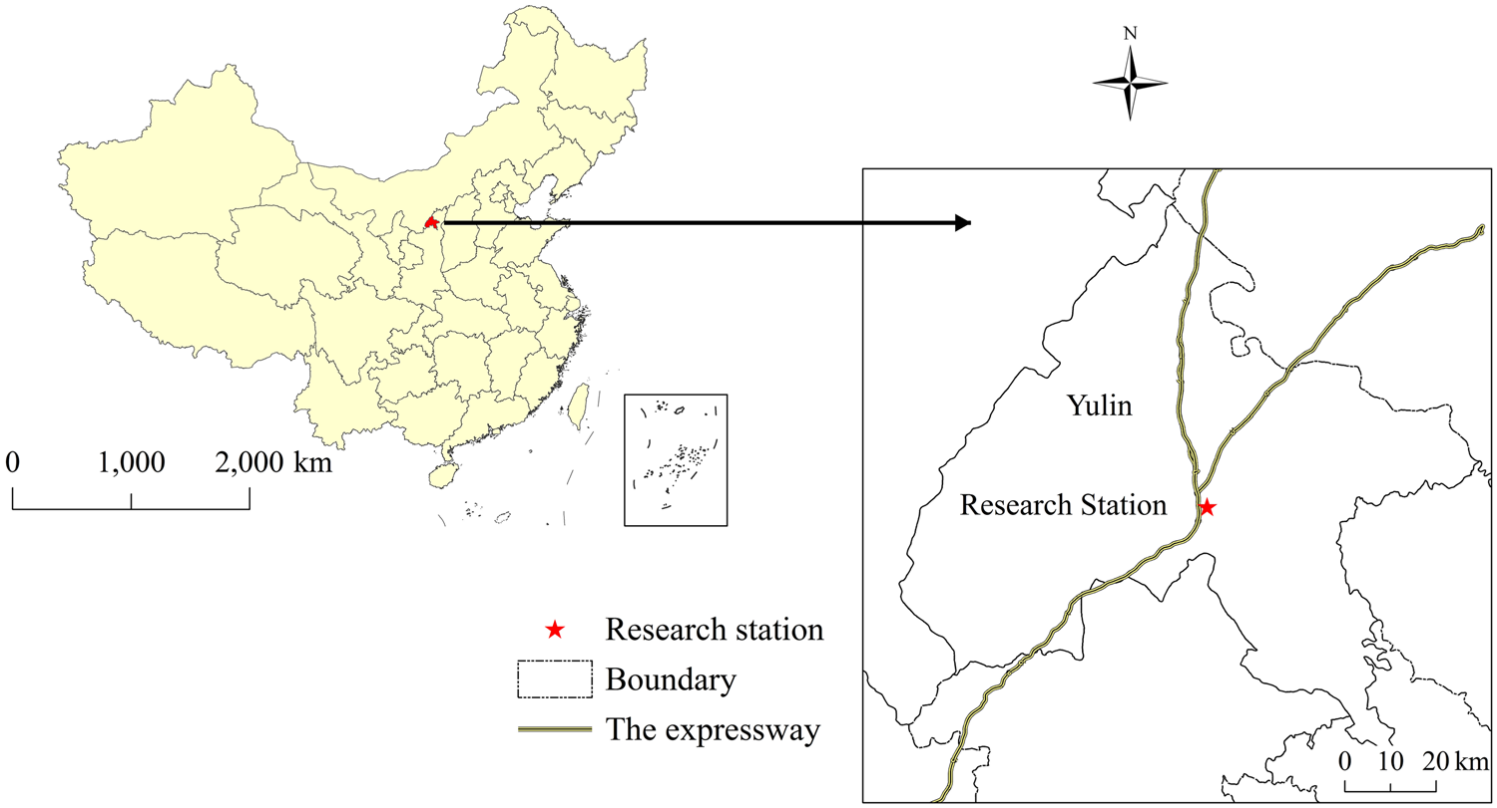
Geographical position of the study area. Map was generated using ArcGIS 9.3 (http://www.esrichina.com.cn/), 1:16, 000, 000 scale map of P.R. China was obtained from National Administration of Surveying, Mapping and Geoinformation (http://bzdt.nasg.gov.cn/), the figure was generated by the procedure of geography correction, map projection transformation, image vectorization, duplicate layers, adding map elements, and outputting the image in TIFF format.

The Research Station (study site) is located on the Rare Psammophytes Protection Botanical Base (RPPBB) in Yulin City, which is the northernmost prefecture-level city of Shaanxi Province (38°20′11″N, 109°42′54″E) (Fig. 6). The study site area was 333.30 ha. The study site has a continental, monsoon-influenced semi-arid climate, with long, cold winters, and hot, humid summers. Annual precipitation is approximately 400 mm. Sunshine is abundant (annual accumulation of 2780 h). The mean annual temperature is 8.8 °C. The frost-free period is approximately 140 d. The RPPBB landscape is characterized by fixed sand dunes, which are classified as arenosol type of quartisamment (U.S. Soil Taxonomy)^21^. The soil pH value is 7.2 ± 0.5, and natural vegetation in the study area consists largely of *Salix psammophila*, *Caragana korshinskii*, *Hedysarum scoparium*, *Artemisia ordosica*, and *Populus alba*.

### Sample plot investigation

The study was conducted from June 2013 to August 2013. A total of 24 MPPs sample plots 20 m × 20 m and with a stand density of 900 ± 5–2700 ± 50 trees.ha^*−*1^ were selected. 8 different density gradients were considered (3 sample plots were taken as reduplicates for each stand density), and each stand density of initial plantation area was 100 m × 100 m; initial planting time was in the year of 1989 (immature timber). These sample plots that were intact and unaffected by human disturbance. Within these plots, the dominant vegetation species was *P*. *sylvestris*, and understory species comprised a sparse grass-shrub layer. Herb cover was less than 30%, and the height was lower than 0.6 m. General information about the MPPs is presented in Table 4. Average tree height (H), diameter at breast height (DBH) and canopy size (C) were 10.05 m, 14.56 cm, and 3.14 m, respectively. For each plot, 3 soil sampling profiles (as reduplicates) were selected at random (not taken from the plot edge). Soil samples were collected for 2 layers: the top layer (0–20 cm) and the sub-top layer (20–40 cm). Soil samples of the 2 layers were also collected in the CK.

**Table 4:** General information of the different density of MPPs plots.

Pn	Sd (Trees.ha^−1^)	H (m)	DBH (cm)	H/DBH	Cd (%)	C (m)
P_I_	2700 ± 50	9.79 ± 0.40	11.29 ± 1.39	0.87 ± 0.03	90 ± 3	1.99 ± 0.08
P_II_	2200 ± 25	8.89 ± 0.20	13.00 ± 0.82	0.68 ± 0.02	80 ± 2	3.12 ± 0.03
P_III_	2050 ± 18	10.35 ± 0.50	13.65 ± 0.67	0.76 ± 0.08	76 ± 4	2.50 ± 0.05
P_IV_	1500 ± 22	10.62 ± 0.90	14.51 ± 0.12	0.73 ± 0.02	50 ± 4	2.49 ± 0.02
P_V_	1400 ± 30	8.30 ± 0.30	13.18 ± 0.11	0.63 ± 0.01	45 ± 5	2.68 ± 0.01
P_VI_	1300 ± 38	10.16 ± 0.50	15.17 ± 0.48	0.67 ± 0.01	70 ± 3	4.07 ± 0.04
P_VII_	1250 ± 8	12.06 ± 0.10	19.04 ± 0.53	0.63 ± 0.03	75 ± 6	4.06 ± 0.03
P_VIII_	900 ± 5	10.26 ± 0.30	16.67 ± 0.16	0.62 ± 0.16	65 ± 5	4.19 ± 0.06

Pn is the plot number, Sd is the stand density, H is the height, DBH is the diameter at breast height, H/DBH is the ratio of diameter at breast height to height, Cd is the canopy density, and C is the canopy size. Values in the parentheses indicate standard error (n = 3).

### Soil fractal model descriptions and measurements

To measure the topsoil particles and fractal characteristics, unscreened air-dried soil samples were pretreated with a hydrogen peroxide solution (30%, w.w^−1^) to eliminate organic matter. Then, the soil aggregates were dispersed by adding sodium hexametaphosphate and sonicating the samples for 30 s^18^. The pretreated soil samples were then analyzed using Malvern MasterSizer 2000 (Malvern Inc. England, UK), which uses a laser diffraction technique with a measurement range of 0.02–2000 mm and a margin of error of 2%^18^. Each sample was measured 5 times and the mean values were calculated. The analysis results of soil PSD were outputs using U.S. Soil Taxonomy as follows: 0–2 μm, 2–50 μm, 50–100 μm, 100–250 μm, 250–500 μm, 500–1000 μm, and 1000–2000 μm^19, 73^.

*D* of soil PSD was calculated as follows (Eq. 1):

1 $$\frac{V(r < Ri)}{VT}={(\frac{Ri}{R{\rm{\max }}})}^{3-D}$$

where *r* is the soil particle size, *R*_*i*_ is the soil particle size of grade *i*, *R*_max_ is the maximum value of soil particle size, *V*(*r* < *R*_*i*_) is the volume of soil particle size less than *R*_*i*_, and *V*_T_ is the total volume of soil particles^21, 23, 25, 30^.

### Methods for soil property analysis

All the soil samples were dried naturally in the laboratory for 2 d. We carefully removed all plant stems, roots and tiny gravels, and then parts of the air-dried soil samples were hand sieved through 2.00 mm and 0.25 mm screens prior to laboratory analysis^21^.

Soil physical properties were analyzed using the following methods: (1) C_P_ and SMC were measured through introduction of ring sampler; (2) T_P_ was calculated using Eq. 2:

2 $${\rm{TP}}=(1-\frac{{\rm{BD}}}{\rho s})\times 100$$

where T_P_ is the total porosity (%), BD is soil bulk density (g.cm^*−*3^), and *ρ*_s_ is soil particle density which is equal to 2.73 g.cm^−3^.

BD was measured using the wax seal method (Eq. 3):

3 $${\rm{BD}}=\frac{100g1}{[(g4-g3)/\rho 1-(g2-g1)/\rho 2]\times (100+W)}$$

where *g*_1_ is the sample weight (g), *g*_2_ is sample weight when completely wrapped by wax, *g*_3_ is the original reading of electronic balance (g), *g*_4_ is reading of electronic balance with the sample (g), *ρ*_1_ is specific gravity of water (equal to 1.0 g.cm^*–*3^) and *ρ*_2_ is specific gravity of wax (equal to 0.9 g.cm^*–*3^)^21^.

Soil chemical properties were analyzed through the following: (1) potassium dichromate wet combustion method for SOC; (2) micro-Kjeldahl’s method for N_T_; (3) Mo-Sb colorimetric method for P_T_; (4) hydrofluoric and perchloric acid (HF-HCLO acid)-flame photometer method for K_T_; (5) alkali diffusion method for N_Avi_; (6) sodium bicarbonate (NaHCO_3_) digestion-Mo-Sb colorimetric method for P_Avi_; and (7) ammonium acetate digestion-flame photometer method for K_Avi_^21^.

### Statistical analysis

Data were analyzed using SPSS software version 21.0 (IBM Inc. NC, USA). The differences in selected soil physicochemical properties and *D* values among the MPPs were compared using multiple comparison and one-way analysis of variance. A least-significant difference test (at *p* < 0.05) was used to compare the means of soil variables. Pearson’s correlation coefficient and a two-tailed test were used to distinguish correlation (significantly correlated at *p* < 0.05 (0.05 level) and *p* < 0.01 (0.01 level)) and significant differences (at the 0.05 level and 0.01 level). Simple linear regression and correlation analysis were performed using OriginLab OriginPro 9.0 software (OriginLab Inc., Northampton, MA, USA) to identify the relationships between *D* and the selected soil properties and stand density (at the 0.05 level and 0.01 level). Data processing and plotting were also completed using OriginLab OriginPro 9.0 software.
